# Severe chronic abuse of zolpidem for over 10 years: a case report and review of similar cases

**DOI:** 10.3389/fpsyt.2023.1252397

**Published:** 2023-09-27

**Authors:** Reza Moshfeghinia, Kimia Jazi, Shabnam Kabaranzadghadim, Mahdi Malekpour, Bahare Oji

**Affiliations:** ^1^Student Research Committee, Shiraz University of Medical Sciences, Shiraz, Iran; ^2^Research Center for Psychiatry and Behavior Science, Shiraz University of Medical Sciences, Shiraz, Iran; ^3^Student Research Committee, Faculty of Medicine, Medical University of Qom, Qom, Iran; ^4^Department of Biochemistry, Faculty of Medicine, Urmia University of Medical Sciences, Urmia, Iran

**Keywords:** zolpidem, high dose, abuse, addiction, dependence

## Abstract

**Background:**

Insomnia is a major health issue, and zolpidem is an effective treatment for insomnia. However, high doses of zolpidem can cause dependence, abuse, and withdrawal symptoms, questioning its advantages.

**Case presentation:**

A 39-year-old woman who has been divorced and unemployed for 2 years was referred to an addiction treatment center with a chief complaint of “seizure-like withdrawal symptoms after consuming high doses of zolpidem (up to 6,000 mg per day) for a decade.” These symptoms were in the form of body tremors, nystagmus, stress, anxiety, hot flashes, and sweaty palms. She has been undergoing detoxification by clonazepam for almost 2 months. Except for the first few days, she did not have any withdrawal symptoms, and her insomnia caused by zolpidem has improved.

**Conclusion:**

Chronic abuse of zolpidem can cause dependence, withdrawal symptoms, and abuse. High doses can lead to extreme cravings and dependence. Physicians must manage the withdrawal process.

## 1. Introduction

Insomnia, a condition that significantly affects the quality of life, is a major public health concern. Insomnia is considered a potential risk factor for diabetes, obesity, cardiovascular diseases, depression, and anxiety. Due to insomnia’s remarkable adverse effects on health, daily performance, and productivity, insomnia not only causes personal irritability but also social impairment (1). Difficulty initiating and maintaining sleep, in addition to waking early in the morning for at least three nights a week in 3 months is classified as insomnia, according to the Diagnostic and Statistical Manual of Mental Disorders fifth edition (DSM-V) (2). Regarding the remarkable adverse effects of insomnia on health, daily performance, and productivity, insomnia not only causes personal irritability but also social impairment (1, 3). Thus, accelerating treatment is of high importance. Cognitive behavioral therapy is known the first line of intervention, while high costs force practitioners to jump into pharmaceutical treatments (4). Of note, several mental and neurological disorders such as anxiety disorders, depression, and mood disorders that are comorbid with insomnia require further intervention (5–7). More importantly, psychological causes and comorbidities such as obsessive worry for return of sleep disturbances strongly influence patients’ compliance to reduce doses and could even induce addiction (8).

In 1992, zolpidem was approved to treat acute insomnia due to its rapid action and few side effects in comparison to benzodiazepines (9, 10). This imidazopyridine or non-benzodiazepine (nBZ)-benzodiazepine agonist attaches selectively to Gamma-aminobutyric acid (GABA)_A_ a1 subunit within the recommended dose of 5–10 mg per day (11). Zolpidem has narcotic effects by binding to the omega 1 (ω1) and omega 2 (ω2) subtypes, resulting in anxiolytic effects, psychomotor impairment, respiratory depression, and anti-epileptic outcomes (12). Zolpidem is a short-acting medication and due to its short half-life, no considerable effects remain the next day. These short acting effects could be due to its fast metabolism by cytochrome P450-3A4 (13).

Excessive sleep, hallucinations, drowsiness, dizziness, headache, and complex nocturnal behaviors are common adverse effects of standard dose uptake of zolpidem (11). A meta-analysis on patients suffering from insomnia disorder approved the safety of zolpidem with no significant adverse events for 1 month (14). However, in higher doses (above 600 mg daily), zolpidem acts unselectively on a_1_, a_2_, a_3_, and a_5_ receptors unselectively. Also, mutations causing lower affinity of the a_1_ subunit with zolpidem, resulting in psychostimulant effects including euphoria, high frequency of talking, sociability, as well as delusions, mania, and psychotic experiences (15–17). Consequently, long-term high doses of zolpidem are highly associated with extreme cravings along with dependence and abuse. Withdrawal symptoms such as weakness of limbs, headaches, considerable irritability, inability to concentrate, tremors, sweating, palpitation, as well as seizures may occur during zolpidem overuse, calling into question the advantages of this medication (15, 18). To date, there have been reports of extremely high daily doses of zolpidem overuse with doses up to 2,400 mg being among the highest (19, 20). Herein, we report a 39-year-old woman presenting with 10 years of chronic zolpidem abuse: up to 6,000 mg/day as the first case with the highest level of consumption (>2,400 mg/day). We also reviewed the reports of previous extreme zolpidem abuse (>1,000 mg/day), focusing on abstinence symptoms to not only guide physicians to identify the withdrawal process but also help them perfectly manage a case of abuse.

## 2. Case presentation

This case report presents a 39-year-old woman with a history of long-term zolpidem abuse and subsequent withdrawal symptoms. The patient was referred to an addiction treatment center by her family and friends during the first 24 h after she stopped taking zolpidem due to seizure-like withdrawal symptoms, which included body tremors, nystagmus, stress, anxiety, hot flashes, and sweaty palms. Additionally, the patient reported experiencing seizures, low self-confidence, feeling of worthlessness, and headaches since 2011 whenever she was off the medication.

The patient’s first exposure to zolpidem occurred 12 years ago after she visited a psychiatrist due to anxiety, hand and jaw tremors, nystagmus, and fainting after short conversations following family problems and arguments. The psychiatrist prescribed 5 mg per night of zolpidem, along with sodium-valproate and alprazolam. Within a year, the patient’s psychiatrist increased the dose of zolpidem to 10 mg per night. However, the patient stops using the rest of the drugs but continued to take zolpidem in excess due to the feeling of peace, carelessness, forgetfulness of stress and problems, feeling of disconnection from the world, and lack of feelings of loneliness. Subsequently, the patient increased her own consumption of zolpidem citing euphoria, happiness, and decreased interest in other issues and relationships.

The patient was slowly increasing the dose of zolpidem, but in 2016, she increased her zolpidem consumption to 300 tablets of 10 mg of zolpidem at once every day and slept for 45 min but reported enjoying the pleasure of sleep during this short sleep duration. The patient used 3,000 mg of zolpidem during the day (mostly during the night), obtained through multiple illegal centers and using fake prescriptions. In Iran, zolpidem must be obtained using a medical doctor’s prescription. The patient abused zolpidem at night and about half an hour after consumption fell asleep for about 45 min. This sleep was highly refreshing, and the patient reported feeling good afterwards. However, she mentioned that she experienced imbalance and blurred vision after waking up. Following her marriage in 2016, the patient reduced her zolpidem consumption to 1,500 mg per day but increased the usage of zolpidem again to 5,000 mg per day after 1 year due to the deterioration of her married life. After divorce in 2019, the patient increased her consumption to 6,000 mg/day (600 tablets per day), which interfered with her ability to work and communication with others. She reported experiencing symptoms such as depression, irritability, anxiety, decreased appetite, and amnesia as well as imbalance 1 h after taking zolpidem. The patient abused this large amount of zolpidem during the day and night in multiple divided doses by consuming large amounts of tablets using spoon or her hand and water; however, she did not mention crushing of the tablets. In some situations, her sister and her niece helped her to peel the pills. The patient’s appetite became worse after abusing this number of pills, and she did not eat food hours before and after consuming the pills. She often used tea and sugar loafs instead of food. Because of the low food intake, she had to go to the clinics to receive serum injections. She mentioned that she could eat this large number of pills because they were tasteless and caused no gastrointestinal adverse effects like nausea, vomiting, or abdominal pain.

The patient attempted to quit zolpidem once in 2022 and was able to abstain for 3 months with the help of admission to a psychiatric ward. During these 3 months without zolpidem, her sleep improved, and her social interactions became relatively normal. However, she ultimately abused zolpidem again and increased her zolpidem consumption to 6,000 mg/day. She started to abuse zolpidem again because she was unemployed and memories of her divorce were bothering her. She later increased the zolpidem abuse to 6,000 mg/day before reducing it again to 4,000 mg/day due to financial constraints. In the last few months of taking zolpidem, the patient unsuccessfully attempted to replace it with opium. After 2 years of being unable to work, the patient was forced to quit taking zolpidem because she had depleted her savings. One day after discontinuing zolpidem, she presented to an addiction treatment center with the help of her friends and family due to experiencing withdrawal symptoms such as body tremors, hot flashes, nystagmus, and sweaty palms.

Upon admission, despite severe and long-term zolpidem consumption, the patient had no other medical issues, such as liver or kidney damage; zolpidem was discontinued, and the patient underwent detoxification with diazepam (20 mg three times a day). The patient experienced seizure like symptoms such as nystagmus and flushing only for the first 2 days. The detoxification medication was changed to clonazepam (41 mg per day in two divided doses) along with escitalopram, quetiapine, and sodium-valproate. Finally, the patient’s condition stabilized with 41 mg of clonazepam. We tapered clonazepam with a taper rate of 10–25% every 3–4 days depending on the patient’s tolerance, and after about 60 days clonazepam was discontinued. Since then, it has been 2 months, and her general condition has improved with the replacement therapy of other drugs. During the detoxification period, her sleeping habits improved, and she sleeps for almost 4 h. Her appetite also became better after zolpidem. Her communication with her family and friends has also improved, and during her hospitalization, her memory has improved.

## 3. Discussion

To the best of our knowledge, this is the first case that reports such a high amount of zolpidem abuse to date. This patient used up to 6,000 mg/day of zolpidem for 10 years. Besides classic withdrawal symptoms including hand and jaw tremors, hot flashes, and sweating, interestingly, after withdrawal of the consuming ultra-high doses of zolpidem, she experienced seizure-like symptoms in addition to nystagmus. Additionally, when not taking the medicine, she suffered from feelings of worthlessness, headaches, and loss of self-confidence.

Zolpidem is a nBZ drug used to treat chronic insomnia and sleep initiation impairment by stimulating deep sleep preservation (21). Like all medications, zolpidem also has significant adverse events. Anaphylaxis, behavioral alterations, and central nerve system depression have been reported as adverse zolpidem effects (22). However, the most important complication of zolpidem is dependency and severe abuse followed by withdrawal symptoms. Previous studies mentioned inability to concentrate, lightheadedness, decreased sleep, sweating, marked irritability, palpitations, nausea, and dysphoria as abstinence symptoms (19, 20, 23). A report of a 20-year-old man with 1,200 mg/day abuse also demonstrated dysarthria due to excessive tension as a possible complication of zolpidem (24). Mao et al. demonstrated withdrawal symptoms chronologically (25). The authors stated that rebound insomnia, cravings, anxiety, and influenza-like symptoms appeared initially from the first days of withdrawal. Following this, skin paresthesia, tonic-colonic-type seizures, and hallucinations appeared on days 8 to 12 of admission (25). Consistently, our 39-year-old patient experienced hand and body tremors, decreased sleep, irritability, cravings, and sweating in addition to episodes of hot flushes. Our patient also experienced seizure-like withdrawal symptoms. Previously, three studies reported seizures as high-dose adverse abuse events (12, 26, 27). The first case was a 34-year-old woman with two seizure-like attacks: one after the first time that she had not taken the medication after 2 years of 2000 mg/day abuse and the second 3 months later (26). The second case was a 49-year-old woman who had two consecutive seizures of partial epilepsy at the right upper limb soon after consuming insufficient dosage (27). The third did not mention any particular data about the seizure (12). However, both low- and high-dose abuse of zolpidem showed seizure (25–27). Our patient also reported two attacks of tremors and hot flushes with an inability to move and palmar sweating with normal levels of consciousness during the detoxification period. She also had a sense of pupillary twitching, uncontrolled movements of the eyes, and jaw locking during the seizure-like episodes.

To date, there has been reports of vision hallucinations and macropsia after single standard zolpidem dose ingestion, which were resolved after consumption was discontinued, and also as a late symptom of drug abuse (25, 28). However, to the best of our knowledge, this is the first report of nystagmus as a withdrawal symptom for zolpidem abuse. Although the reason is unknown, being female and having malnutrition, which can cause low albumin concentrations, have been recommended as possible predisposing factors (29). It is believed that the zolpidem concentration in the blood is higher in women, and since zolpidem binds to albumin in plasma, lower levels of zolpidem result in higher active zolpidem concentrations (30). Consequently, in our case, in addition to being female, high-dose zolpidem abuse could be an explanation for nystagmus as a novel withdrawal symptom; nevertheless, further pharmacological studies are highly recommended.

A previous case report highlighted delirium as a novel withdrawal symptom in low-dose zolpidem uptake concomitant with alcoholism (31). An important challenge in prescribing the medication is the effect of other concomitant medications on cytochrome P450 (CYP450), which metabolizes zolpidem. A case report highlighted the possibility of ultra-high levels of zolpidem to alter the activity of CYP450, which causes manifold plasmatic concentrations despite having a neutral effect on the enzyme in therapeutic dosage (17). This finding highlights the need for further evaluations of drug history and cautious zolpidem prescription to avoid threats of poly-consumption.

To the best of our knowledge, this is one of the first times that single therapy with clonazepam has been used without continuing a tapering dose of zolpidem or any other medication concomitantly. This patient showed nystagmus and redness of the eye during the first 2 days of therapy, and further hot flash attacks were revealed with diazepam injection. Confirming our decision, Bajaj et al. stopped zolpidem and started titrating doses of clonazepam and propranolol to treat the abstinence symptoms for 10 days (19). Furthermore, to detoxify zolpidem in a 29-year-old man with 1,200 mg/day abuse, authors implied a combination therapy of tapering zolpidem (starting with 10 mg daily), chlorpromazine, and 6 mg clonazepam (24). Another study gradually replaced tapering zolpidem with sertraline, pregabalin, and clonazepam in a case of 1,200 mg daily abuse (32). There have also been various detoxifying regimens; single pregabalin therapy, diazepam in addition to trazodone and propranolol, or bupropion and mirtazapine, which can be seen in Table 1 (20, 26, 27). To the best of our knowledge, as with benzodiazepines (33), there is no documented guideline to approach zolpidem abuse and patients are conditionally treated. The efficacy of substituted medications is vague. Of note, further studies should focus on the association between pharmaceutical detoxification and the severity of withdrawal symptoms. Additionally, the relationship between treatments and the possibility of re-abuse of zolpidem is also of high priority to evaluate.

**Table 1:** Characteristics of cases reported with more than 1,000 mg zolpidem consumption (M stands for male and F stands for female).

References	Country	Sex/Age	Psychiatric diagnosis	Maximum doses (mg/day)	Drugs in combination	Duration of zolpidem use (month)	Withdrawal symptoms	Management
Bajaj et al. (19)	India	M/45	Alcohol abuse Insomnia	2,400	Lorazepam, supportive treatment for detoxification of alcohol	60	Weakness of limbs, inability to concentrate, light, headedness, decreased sleep and marked irritability	Clonazepam, Propranolol
Huang et al. (26)	Taiwan	F/34	Adjustment disorder with depressed mood Insomnia	2000	None	24	Anxiety, hand tremor, sweating, and palpitations, seizure	Diazepam, Propranolol, Trazodone
Lyu et al. (20)	China	F/23	Insomnia	1,400	None	72	Tremor, sweating, palpitation, nausea and dysphoria	Diazepam, Bupropion, Mirtazapine
Chattopadhyay et al. (23)	India	M/33	Zolpidem was taken for euphoria	1700	Quetiapine	60	Minor headaches, mild tremors of hands	Not mentioned
Ohshima et al. (24)	Japan	M/29	Insomnia	1,200	Quazepam, Brotizolam	60	Dysarthria due to excessive tension/ Irritability	Zolpidem, Chlorpromazine, Clonazepam
Oulis et al. (27)	Greece	F/49	Dysthymic disorder Insomnia	1,500	Several antidepressant agents	60	Two consecutive seizures of partial epilepsy	Pregabalin
Chiaro et al. (32)	Switzerland	F/70	Posttraumatic stress disorder Insomnia	1,200	None	Not mentioned	Not mentioned	Sertraline, Clonazepam, Pregabalin
Djezzar et al. (17)	France	M/38	Not mentioned	1,000	Dextromoramide, Methadone	Not mentioned	Sweating and general malaise, seizure	Not mentioned
Heydari et al. (12)	Iran	M/32	Tic anxiety	2000	Heroin, Diphenoxylate	24	Classic symptoms and signs of opioid withdrawal (runny nose, diarrhea, myalgia, irritability, insomnia, …), convulsion	Not mentioned

Despite the patient’s depression, irritability, anxiety, amnesia, diplopia, and imbalance after drug intake, our 39-year-old patient continued zolpidem consumption for the consequent feelings of euphoria, happiness, and a state of ignorance of life’s issues that zolpidem induced. Initially, our patient found zolpidem significantly effective in reducing her anxiety and aforementioned physical symptoms. At the same time, she reported better sleep quality and quantity in the first years of consumption. Increasing the dose, disregarding her clinician, she also stated that she could not sleep well. The sleep, anxiety, and physical symptoms were not the problem anymore. Worthlessness, loss of self-confidence, and the pressure of her life’s issues forced her to continue taking higher and higher doses of zolpidem for relief. Despite the mental relief, higher doses affected her physically, which forced her to take higher doses to achieve both mental and physical relief. Combination therapy would be a wise choice in such cases. Furthermore, familial status could significantly affect the therapeutic decision. Our patient stated that her husband was the cause of her anxiety, and the initial zolpidem abuse was to get rid of this negative effect on her life. During these 12 years of zolpidem use, she got divorced and was left alone with no job and no support. Following this, she continued using higher doses to avoid the feelings of emptiness. Therefore, drugs such as zolpidem, with its high probability of abuse and consequent withdrawal symptoms, should be prescribed for patients who have emotional support and engagement whether it is through family or personal care. Beyond this, psychotherapy was not effective, and the patient would not continue without proper support. Besides, considering patients have reported experiencing neurological symptoms, such as fainting and nystagmus during their disease, we suggest that zolpidem could not be the choice therapy, as there is a high probability of abuse. Numerous cases of zolpidem abuse, specifically with ultra-high doses, bring about further concerns of possible irreversible withdrawal symptoms and unknown future episodes. Disregarding classic symptoms, there could also be other possible emergent symptoms, including tongue and larynx angioedema in higher-dose abuse, that physicians should estimate and consider (22). There remain challenges with treatment, and we suggest future investigations should focus on the efficacy of pharmaceutical therapy on each symptom and treatment response.

In conclusion, chronic abuse of zolpidem is a serious problem that can lead to withdrawal symptoms, dependence, and abuse. Zolpidem is an effective short-term medication for insomnia when used within recommended doses; however, high doses of zolpidem may result in psychostimulant effects, leading to extreme craving and dependence. Although the adverse events are rare and many patients benefit from Zolpidem, it is highly important to prevent potential irreversible dependence. New sale and prescription policies in addition to combination therapy could be considerable solutions.

This case report highlights a 39-year-old woman with 12 years of chronic zolpidem abuse, with the highest level of consumption at over 6,000 mg/day, for the first time. The case emphasizes the importance of preventing, identifying, and managing the withdrawal process and offers guidance for physicians. This study had strengths: while many zolpidem-dependent patients suffer drug and alcohol abuse simultaneously, our patient was a single zolpidem dependent, which helped us follow only novel withdrawal symptoms in high doses of abuse. In addition, we did not taper zolpidem even though there were high concentrations of abuse. Certainly, this case report also had limitations. First, our patient had a history of nystagmus and tremors according to her anxiety. Although the patient experienced exacerbation of symptoms accompanied by further withdrawal symptoms such as seizure-like symptoms and hot-flashes, it was difficult to prove that symptoms were not caused by the clinical deterioration of anxiety. Second, the blood test for zolpidem concentration was not taken when the patient was admitted to assess the amount of zolpidem abuse; however, it is noteworthy that not only the patient but also her sister and the pharmacy where she used to buy the medications approved the exact amount of abuse. Moreover, her sister found the empty blister packs. Third, drug abuse as a chronic recurring process requires a chronic follow-up plan to evaluate the therapeutic effects of treatment and prevent the reoccurrence of abuse. Future evaluations should also mention longer follow-up intervals.

## Data availability statement

The original contributions presented in the study are included in the article/supplementary materials, further inquiries can be directed to the corresponding author.

## Ethics statement

The studies involving humans were approved by Ethics committee of Shiraz University of Medical Sciences. The studies were conducted in accordance with the local legislation and institutional requirements. Written informed consent for participation in this study was provided by the participants’ legal guardians/next of kin. Written informed consent was obtained from the individual(s), and minor(s)’ legal guardian/next of kin, for the publication of any potentially identifiable images or data included in this article.

## Author contributions

BO, RM, and SK managed the patient and collected the data in cooperation with MM. RM and KJ drafted the manuscript and reviewed the literature. BO supervised the project. All authors reviewed successive drafts, contributed to the article, and approved the submitted version.

## Conflict of interest

The authors declare that the research was conducted in the absence of any commercial or financial relationships that could be construed as a potential conflict of interest.

## Publisher’s note

All claims expressed in this article are solely those of the authors and do not necessarily represent those of their affiliated organizations, or those of the publisher, the editors and the reviewers. Any product that may be evaluated in this article, or claim that may be made by its manufacturer, is not guaranteed or endorsed by the publisher.
